# Clinical and radiomic features for predicting the treatment response of repetitive transcranial magnetic stimulation in major neurocognitive disorder: Results from a randomized controlled trial

**DOI:** 10.1002/hbm.26032

**Published:** 2022-08-01

**Authors:** Hanna Lu, Sandra Sau Man Chan, Sukling Ma, Cuichan Lin, Vincent Chung Tong Mok, Lin Shi, Defeng Wang, Arthur Dun‐Ping Mak, Linda Chiu Wa Lam

**Affiliations:** ^1^ Department of Psychiatry The Chinese University of Hong Kong Hong Kong SAR China; ^2^ The Affiliated Brain Hospital of Guangzhou Medical University Guangzhou China; ^3^ Department of Medicine and Therapeutics The Chinese University of Hong Kong Hong Kong SAR China; ^4^ Department of Imaging and Interventional Radiology The Chinese University of Hong Kong Hong Kong SAR China

**Keywords:** cortical thickness, depression, neurocognitive disorder, radiomics, randomized controlled trial, transcranial magnetic stimulation

## Abstract

Image‐guided repetitive transcranial magnetic stimulation (rTMS) has shown clinical effectiveness in senior adults with co‐occurring depression and cognitive impairment, yet the imaging markers for predicting the treatment response are less investigated. In this clinical trial, we examined the efficacy and sustainability of 10 Hz rTMS for the treatment of depression and cognitive impairment in major neurocognitive disorder (NCD) patients and tested the predictive values of imaging‐informed radiomic features in response to rTMS treatment. Fifty‐five major NCD patients with depression were randomly assigned to receive a 3‐week rTMS treatment of either active 10 Hz rTMS (*n* = 27) or sham rTMS (*n* = 28). Left dorsolateral prefrontal cortex (DLPFC) was the predefined treatment target. Based on individual structural magnetic resonance imaging scans, surface‐based analysis was conducted to quantitatively measure the baseline radiomic features of left DLPFC. Severity of depression, global cognition and the serum brain‐derived neurotrophic factor (BDNF) level were evaluated at baseline, 3‐, 6‐ and 12‐week follow‐ups. Logistic regression analysis revealed that advanced age, higher baseline cognition and randomized group were associated with the remission of depression. Increased cortical thickness and gyrification in left DLPFC were the significant predictors of clinical remission and cognitive enhancement. A 3‐week course of 10 Hz rTMS is an effective adjuvant treatment for rapid ameliorating depressive symptoms and enhancing cognitive function. Pre‐treatment radiomic features of the stimulation target can predict the response to rTMS treatment in major NCD. Cortical thickness and folding of treatment target may serve as imaging markers to detect the responders. ChiCTR‐IOR‐16008191, registered on March 30, 2016.

## Graphical abstract

A 3‐week course of 10 Hz repetitive transcranial magnetic stimulation (rTMS) is an effective treatment for ameliorating depressive symptoms and enhancing cognitive function. Pre‐treatment radiomic features of the stimulation target can predict the responses to rTMS treatment in major NCD. Cortical thickness and folding of treatment target may serve as an imaging marker to detect the responders.

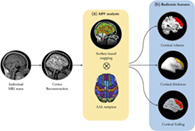

## 1 INTRODUCTION

In the context of rapid population ageing, the burden of dementia is usually compounded by the presence of co‐occurring neuropsychiatric symptoms, of which depression is one of the prominent and distressing symptoms to daily life (Jia et al., 2018; Wise et al., 2019). Epidemiological study showed that more than 60% of the senior adults in Hong Kong are suffering from the co‐existence of cognitive dysfunction and depression (Fung et al., 2017). When focusing on clinical populations, meta‐analyses have reported that the symptoms of depression are present in nearly 25% of patients with mild neurocognitive disorder (NCD) and have a severe impact on the cognitive function and quality of life in dementia patients (Asmer et al., 2018; Ismail et al., 2017). Considering depression can speed up the progression of cognitive impairment and deteriorate the quality of life (Saczynski et al., 2010), there is both a clinical unmet need and an opportunity to take advantage of technological advances to optimize and personalize the management of co‐occurring cognitive impairment and depression in senior adults.

Image‐guided transcranial magnetic stimulation (TMS) is a non‐invasive technology that has attracted considerable attention as a safe and effective intervention for treating different types of brain disorders, including major depressive disorder (Sayar et al., 2013), Alzheimer's disease (Koch et al., 2020) and stroke (Takeuchi et al., 2005). The putative mechanisms for TMS effects are related to the modulation of cortical activity and neuroplasticity by inducing the flow of electric current at the targeted cortical surface through the powerful and rapidly changing magnetic fields (George et al., 1999). There is preliminary evidence that supports the antidepressant effects of high‐frequency repetitive TMS (rTMS) over left dorsolateral prefrontal cortex (DLPFC) in the patients with vascular depression who are not responsive to pharmacological treatments (Brys et al., 2016; Jorge et al., 2008). On the cellular level, prominent changes of synaptic plasticity with the presence of an increased expression of serum brain‐derived neurotrophic factor (BDNF) after successive rTMS treatment have been observed in rodent models of brain ageing and dementia (Shang et al., 2016). Interestingly, a few studies using rTMS to enhance the cognitive function in dementia patients also observed an improvement in mood (Baruch et al., 2019; Cotelli et al., 2008). However, it should be noted that the individual differences in the treatment of depression and cognitive impairment inferred from previous observational studies and clinical trials can raise significant challenges for interpreting the treatment effects of rTMS. The predominant challenges, including potential placebo effects and the neuroanatomical variances due to age‐related brain atrophy (Kirsch, 2019; Rutherford et al., 2017), should be seriously considered in clinical practice.

Taken together, it remains unclear about the clinical effectiveness of placebo‐controlled rTMS in senior adults with co‐occurring depression and cognitive impairment. Therefore, the main purpose of this study was to evaluate the safety, efficacy, and sustainability of a 3‐week 10 Hz rTMS treatment in the management of depression in major NCD patients. Secondly, we proposed to examine whether pre‐treatment clinical and imaging features can predict the rTMS effects on depression and cognitive function during a 2‐month follow‐up period with separate models.

## 2 METHODS

### 2.1 Study design and participants

**FIGURE 1:**
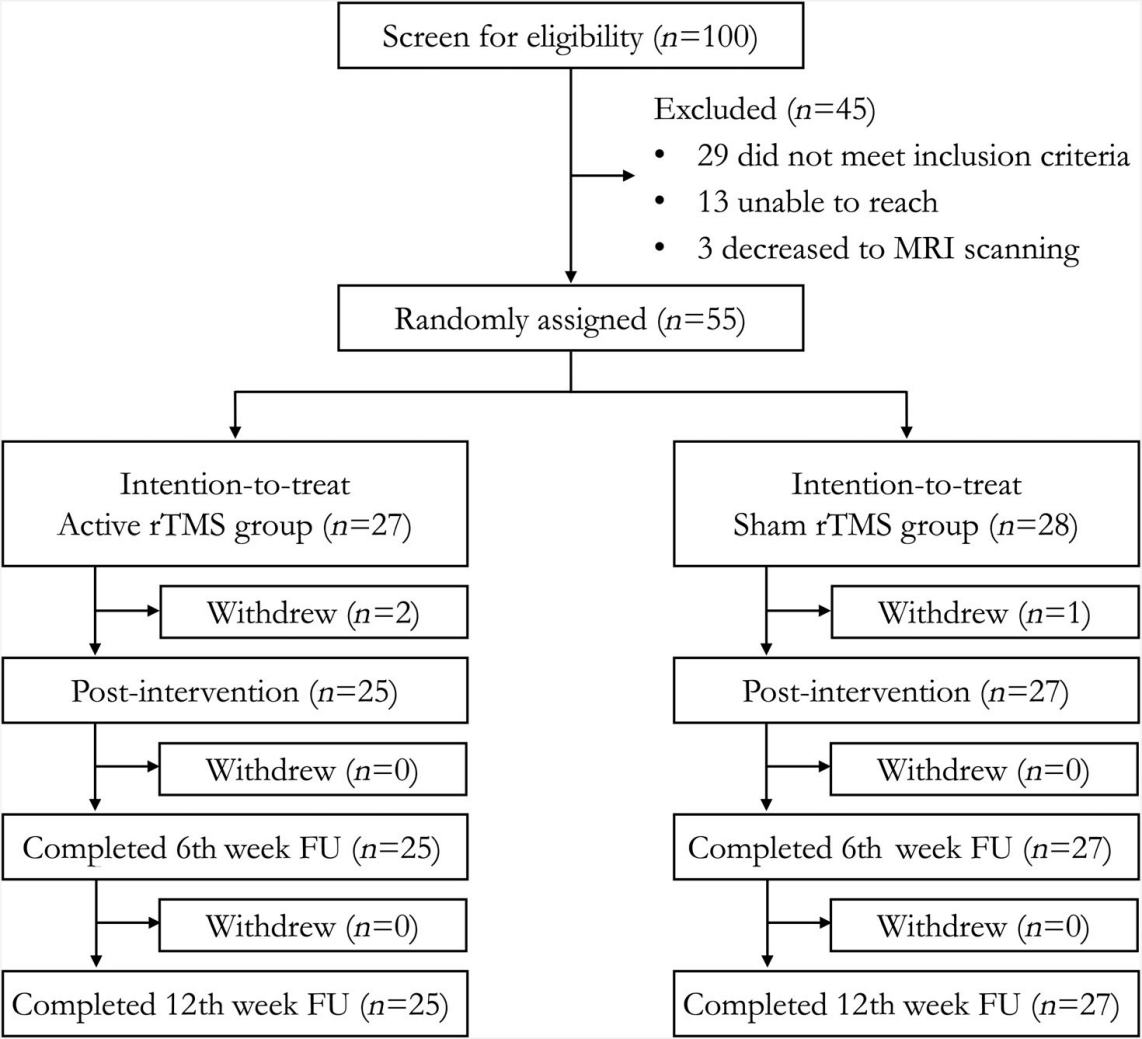
The CONSORT flow diagram for the neuronavigated 10 Hz rTMS clinical trial. CONSORT, Consolidated Standards of Reporting Trials; rTMS, repetitive transcranial magnetic stimulation

This study was a randomized, single blind, sham‐controlled clinical trial conducted at the Chen Wai Wai Vivien Foundation Therapeutic Physical Mental Exercise Centre, The Chinese University of Hong Kong (Trial Number: ChiCTR‐IOR‐16008191). The logistics of this trial followed the Consolidated Standards of Reporting Trials (CONSORT) flow diagram was depicted in Figure 1. Eligible participants were screened, enrolled and randomly assigned to a 3‐week treatment of either active rTMS or sham rTMS. After the rTMS treatment, all the eligible participants were followed up for 8 weeks.

We considered participants eligible if they satisfied the following inclusion criteria (American Psychiatric Association, 2013): (1) Adults aged from 60 to 90 years; (2) with a DSM‐5 diagnosis of either major NCD due to Alzheimer's disease (NCD‐AD) or major NCD due to vascular disease (NCD‐vascular), including significant cognitive decline in the core domains of cognitive function (e.g., attention, memory, perceptual‐motor, language and executive function), and with clinical or neuroimaging features indicative of either Alzheimer's disease or neurovascular disease; (3) with a rating score of 7 or above in the Cornell Scale for Depression in Dementia (CSDD), indicating clinical depression (Pieper et al., 2016). Any anti‐dementia and other psychotropic medications that the participants took were kept constant and carefully recorded throughout the clinical trial.

We excluded the participants who had a history of neuropsychiatric disorders and major neurological disorders, including stroke, transient ischemic attack and traumatic brain injury. The participants were also excluded if they were unable to complete the magnetic resonance imaging (MRI) scanning and rTMS treatment due to the contraindications (e.g., metal inside or on the body).

### 2.2 Procedure

#### 2.2.1 Randomization and masking

After the baseline assessment, all the eligible participants were randomly assigned to either active rTMS or sham rTMS following a 1:1 ratio without restrictions. The randomization sequence was generated by a statistician who was not involved in the study design using an online system (http://randomization.com/) to allocate 27 NCD patients to active rTMS group (treatment group) and 28 patients to sham rTMS group (control group). The participants and the research staff who conducted the assessments were blinded to the study design and group allocation. It was not possible to blind the research staff and clinician who conducted the rTMS, thus they were reminded not to discuss the group allocation with the participants and the research staff who conducted the assessments.

#### 2.2.2 Pre‐intervention and preparation

Structural MRI scans were acquired using a 3.0 T MRI scanner (Philips Healthcare) at the Prince of Wales Hospital in Hong Kong. The T1‐weighted magnetization prepared rapid gradient echo (MPRAGE) sequence was empirically optimized for the grey‐white contrast, with repetition time (TR) = 7.6 ms, echo time (TE) = 3.5 ms, field of view (FOV) = 230 mm, thickness = 0.6 mm, axial acquisition matrix = 256 × 256 × 192, flip angle (FA) = 15° and the voxel size of 1 mm × 1 mm × 1 mm.

#### 2.2.3 Image‐guided rTMS treatment

The pipeline of image‐guided rTMS treatment contained three main steps: (1) Reconstruction and localization: Based on individual structural MRI scans, we first reconstructed the scalp and cortex, and then localized the stimulation targets labelled with the Montreal Neurological Institute (MNI) coordinates (*x, y, z*) in the neuronavigation system. (2) Motor threshold (MT) measurement: MT was measured through the single‐pulse TMS over the left primary motor cortex (M1, hand area) prior to the first rTMS treatment. The individual MT was defined as the minimum stimulus intensity required to trigger a motor evoked potential (MEP) with a peak‐to‐peak amplitude ≥50 μV in at least 5 out of 10 consecutive trials recorded in the electromyography (EMG) system (Figure 4a) (Fitzgerald et al., 2009). (3) Repeated rTMS treatment: 15 sessions of 10 Hz rTMS over left DLPFC were provided by the Magstim Super Rapid stimulator (Magstim Company Ltd.) that generates short duration biphasic pulses. The Magstim 70 mm figure‐8 coil (D70 AirFilm Coil AFC) was held in place with a custom‐made stand tangential to the scalp with the handle pointing back and away from the midline at 45°.

For each participant, the position and markers on the scalp and cortex were identified using the infrared tracking system (Polaris Northern Digital). We targeted the location of left DLPFC with the MNI coordinates as [*x* = −46, *y* = 45, *z* = 38], being carefully to locate this region within the grey matter on the top of middle frontal gyrus (MFG) in the Brainsight neuronavigation system (Fitzgerald et al., 2009; Fox et al., 2012; Lu, Chan and Lam, 2012). The pipeline of procedure of sham rTMS followed the same steps described above, except that a sham coil was used for the sham rTMS treatment.

The rTMS intensity was set at 120% of MT. One session of rTMS treatment contained 30 trains of TMS with 5‐s 10 Hz stimulation followed by a 25‐s rest period. This protocol resulted in the delivery of 1500 pulses in each session of rTMS treatment. The treatment schedule of this trial was one session per day, 5 days per week, lasting for 3 weeks. In the condition of sham rTMS, same rTMS system (i.e., Magstim Super Rapid stimulator) was used for single blind trial with a sham TMS coil (D70 AirFilm Sham Coil AFC). Sham rTMS involved a clinical replicating the sound of the magnetic discharges in order to compensate for the acoustic and other non‐specific effect, without any magnetic pulse being delivered.

### 2.3 Intervention schedule

Fifteen sessions of 10 Hz rTMS treatment were conducted by trained clinician. Measures of treatment outcomes were collected at four time points, including T0 (baseline), T1 (3rd week, post‐intervention), T2 (6th week, 3 weeks post‐intervention) and T3 (12th week, 6 weeks post‐intervention). All the members of research team were trained to ensure the data quality, accuracy, consistency and completeness.

### 2.4 Structural MRI data analysis

#### 2.4.1 Measures of neurovascular effects on the brain

Based on individual T2‐weighted structural MRI scans, white matter hyperintensities (WMH), including periventricular hyperintensity and deep WMH, are used for quantifying the impact of neurovascular factors on the brain in senior adults (Wardlaw et al., 2015).

#### 2.4.2 Analysis of radiomic features

Radiomics, as a quantitative method for medical imaging, aims at enhancing the existing data available to clinicians by means of advanced computational analysis (van Timmeren et al., 2020). Based on individual structural MRI scans, radiomic features of cortical region were extracted and quantified through atlas‐based volumetry (ABV) analysis using the Statistical Parametric Mapping 12 toolbox (SPM12, Wellcome Trust Centre for Neuroimaging, http://www.fil.ion.ucl.ac.uk/spm/software/spm12/) running on MATLAB (R2021a, The Math Works Inc. ) (Gorges et al., 2020; Huppertz et al., 2010).

**FIGURE 2:**
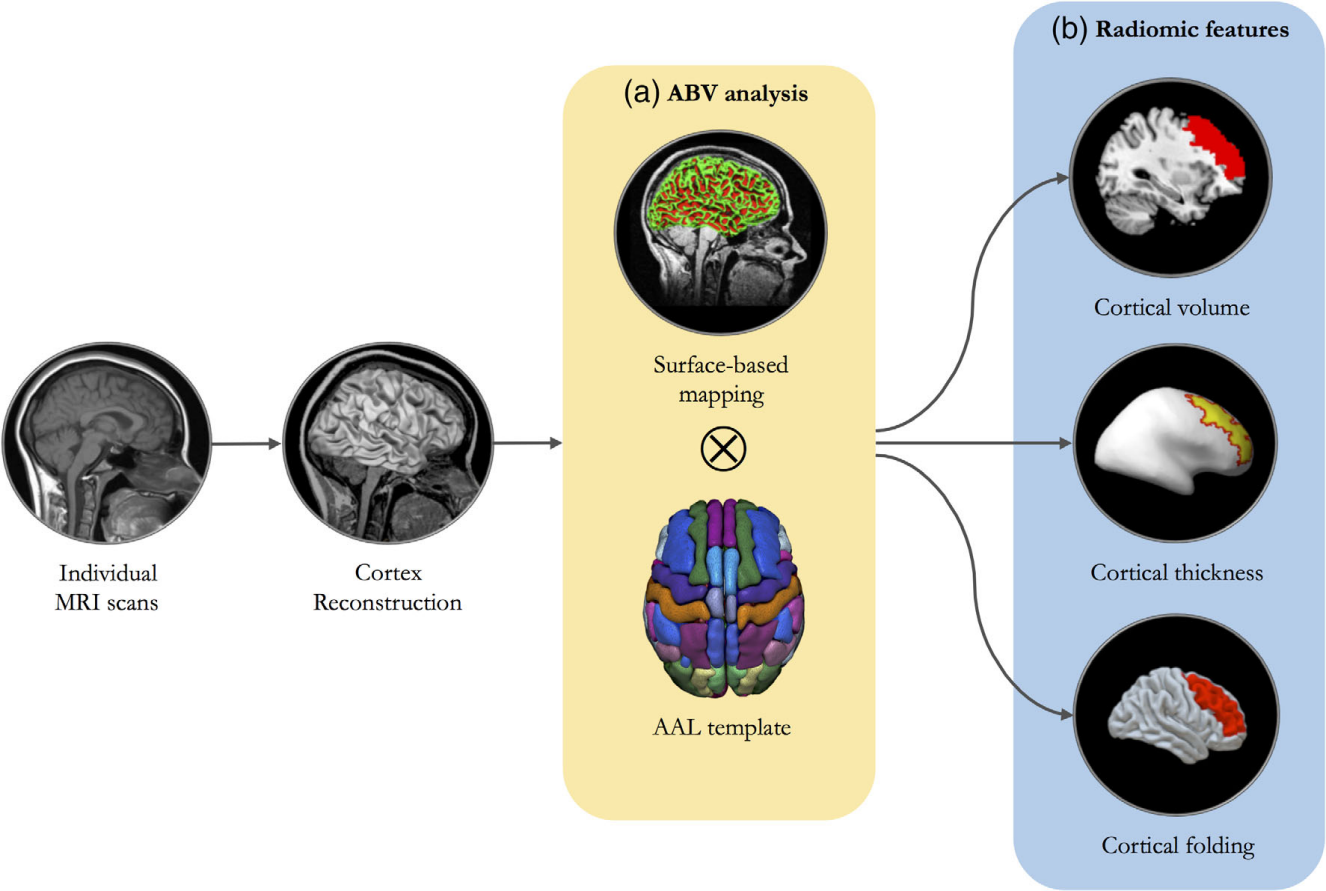
The framework of region‐specific surface‐based analysis of radiomic features. (a) Based on individual structural MRI data, the ABV analysis of cortical surface was performed to quantify the brain structure. (b) Based on the AAL template, the radiomic features of left DLPFC included cortical volume, thickness and folding. AAL, automated anatomical labelling; ABV, atlas‐based volumetry; DLPFC, dorsolateral prefrontal cortex; MRI, magnetic resonance imaging

For this study, left DLPFC, as the predefined treatment target, was the region of interest (ROI) for the analysis of radiomic features. Based on Automated Anatomical Labelling (AAL) template, left DLPFC was verified as MFG (Lu et al., 2021). The GM volume, WM volume and CSF volume of left DLPFC were calculated individually (Figure 2). To adjust the variance of head size, Cendes method was employed to correct the individual variance with total intracranial volume (TIV) through the formula (Cendes et al., 1993): Corrected SV = (MBV × SV)/IBV. MBV is the mean brain volume in the group (a constant), SV is the regional GM volume and IBV is the individual brain volume.

The radiomics of cortical region represent the mathematical features of the reconstructed cortex in three‐dimensional space, including cortical thickness and folding. Cortical thickness is calculated as an average of the distance from the white matter surface to the closest point on the pial surface and from that point back to the closest point to the white matter surface (Vuksanović et al., 2019). Cortical folding, measured by gyrification index (GI), is a ratio of the inner surface area to the area of an outer surface that smoothly encloses the cortex (Cao et al., 2017; Lu, 2020; Madan, 2021).

### 2.5 Measures of treatment outcomes

#### 2.5.1 Primary outcomes

1 Severity of depression:
1 Objective measurement: The CSDD was used for assessing the depressive symptoms in NCD patients, including five domains: mood‐related signs, behavioural disturbance, physical signs, cyclic functions and ideational disturbance (Schreiner et al., 2003). CSDD is a clinician‐rated instrument assessing a range of symptoms associated with depression in dementia and has good sensitivity (0.9) and specificity (0.75). In this study, a cutoff CSDD total score of 7 indicated clinically significant depressive symptoms in NCD patients. The responders were defined as the ones who have a 50% reduction in the CSDD total scores from the baseline to follow‐ups (Lyketsos et al., 2003).
2 Subjective measurement: The Centre for Epidemiologic Studies Depression Scale (CES‐D) is a commonly used self‐reported, psychometric scale intended to identify the frequency and severity of depressive symptoms in a general population (Blodgett et al., 2021). The CES‐D covers four domains, including depressed affect, positive affect, somatic symptoms and interpersonal difficulties (Getnet & Alem, 2019).
2 Global cognitive function: The Montreal Cognitive Assessment Hong Kong version (HK MoCA) was used to measure the global cognitive function and its changes associated with the treatment in senior adults (Wong et al., 2009; Lu, Chan, Chan, Lin, Cheng, and Lam, 2019). HK MoCA is a 30‐point neuropsychological test, covering the main domains of cognitive function, such as attention, visuospatial ability, executive function, short‐term memory and orientation to time and space. HK MoCA was validated in local community‐based population with demonstrated sensitivity to the changes in global cognition in Alzheimer's disease and vascular disease (Lu et al., 2016).

#### 2.5.2 Secondary outcomes

1. Neuropsychiatric symptoms: The Chinese Neuropsychiatric Inventory (NPI) was used to assess the changes of neuropsychiatric symptoms across 12 domains (Wong et al., 2014).
2. Quality of life: The Quality of Life in Alzheimer's disease scale (QoL‐AD), including the patient and caregiver components, is a 13‐item scale designed for measuring the quality of life in dementia patients (Logsdon et al., 1999). A Chinese version of QoL‐AD with demonstrated good psychometric properties was used in this study.
3. BDNF: The level of serum BDNF was used as a peripheral marker of neuroplastic response to the rTMS treatment (Brunoni et al., 2008). Serum BDNF was assayed in duplicate according to manufacturer's instructions (R&D Systems, Inc.).
4. Adverse events: A checklist of potential adverse effects associated with the rTMS administration. The checklist was used to monitor the tolerability and adverse events throughout the repeated sessions of rTMS treatment. The Adverse Event Checklist (AEC) covering the symptoms from eight systems, including body as whole, cardiovascular, digestive, circulatory, urogenital systems, metabolic, musculoskeletal and nervous system was conducted at each assessment points through the study (Lu & Lam, 2019).

### 2.6 Statistical analysis

Baseline demographic and clinical characteristics were compared between the randomized groups using two‐sample *t* test for continuous variables and chi‐squared test for categorical variables. For the treatment outcomes, the score changes of CSDD and HK MoCA over the course of treatment were modelled using a linear‐mixed model (LMM). Treatment (i.e., group allocation), time points and their interactions were modelled as fixed effects. Participants were modelled as random effects at different time points. The score changes of CSDD and HK MoCA and randomized treatment group were tested with occasions (time points) at level one and participants were tested at level two. Cohen's *d* was calculated as the measure of effect size, of which the Cohen's *d* ≥ 0.8 refers to a large effect, 0.5 ≤ Cohen's *d* < 0.8 refers to a medium effect and Cohen's *d* < 0.5 means the effect is statistically significant but small (Monroe et al., 2016).

For the predictive model, we evaluated the demographic, clinical and radiomic features with a plausible theoretical reason to be important (e.g., chronological age), as well as the features previously demonstrated as important in the prediction of response and remission after rTMS treatment. The chosen features included age, sex, years of education, baseline HK MoCA score and the radiomic features of the treatment target (i.e., left DLPFC). Next, the potential predictor variables were included in the logistic regression models to determine their associations with response, remission and cognitive changes as measured by odds ratio (OR) and the corresponding 95% confidence intervals (CI). Because this clinical trial had two randomized groups, we included the group allocation (i.e., active rTMS and sham rTMS) in the model as a covariate. All statistical analyses were conducted using SPSS Statistics 24.0 (IBM). Statistical tests were two‐tailed, with the alpha (type I error rate) set to 0.05.

## 3 RESULTS

### 3.1 Demographics and clinical features

One hundred participants were screened for the eligibility of this clinical trial, of which 45 participants were excluded upon screening and 55 participants were randomly assigned to receive either active rTMS (*n* = 27) or sham rTMS (*n* = 28) (Figure 1). Twenty‐nine (64%) of 45 participants were excluded due to not meeting the inclusion criteria for this study (i.e., the severity of depression), 16 of them (36%) were excluded for declining the MRI scanning.

Baseline demographic features, global cognitive function, severity of depression, regular medication use and global brain morphometry of the participants were comparable between active rTMS and sham rTMS groups (Table 1). The mean age of sham rTMS group (73.7 ± 7.2 years) was older than that of the active rTMS group (69.2 ± 7.3 years) (*t* test, *p* = .02).

**TABLE 1:** Baseline demographic and clinical characteristics in the two randomized rTMS groups

	Active rTMS (*n* = 27)	Sham rTMS (*n* = 28)	*t* value (*χ* ^2^)	*p*
Age	69.2 ± 7.1	73.7 ± 7.2	−2.39	.02
Sex (F/M)	19/8	19/9	0.04	.84
Education (years)	7.3 ± 4.4	7.5 ± 5.2	−0.10	.92
CIRS	4.9 ± 3.0	4.1 ± 1.9	1.17	.25
CSDD	12.7 ± 4.3	11.5 ± 3.7	1.11	.27
CES‐D	21.9 ± 10.8	20.5 ± 12.1	0.46	.65
HK MoCA	20.3 ± 5.3	19.9 ± 6.1	0.29	.78
QoL‐AD	29.4 ± 5.8	27.1 ± 7.4	1.06	.29
CNPI	11.1 ± 8.7	10.6 ± 9.4	0.18	.86

*Note*: Data are raw scores and presented as mean ± SD.

Abbreviations: CES‐D, The Centre for Epidemiologic Studies Depression Scale; CIRS, cumulative illness rating scale; CNPI, Chinese Neuropsychiatric Inventory; CSDD, Cornell Scale for depression in dementia; HK MoCA, Montreal Cognitive Assessment Hong Kong version; QoL‐AD, quality of life in Alzheimer's disease; rTMS, repetitive transcranial magnetic stimulation.

### 3.2 Measures of treatment outcomes

Within each randomized group, significant reductions in the severity of depressive symptoms and improvements in global cognitive function were observed over the follow‐up periods (i.e., T1–T3). As shown in Table 2, the score change of CSDD from baseline to T1 was 3.24 (SE 0.71) in active rTMS group (Cohen's *d* = 0.92; *p* < .001) and 2.15 (SE 0.62) in sham rTMS group (Cohen's *d* = 0.66; *p* = .002). The score change of HK MoCA from baseline to T1 was 1.96 (SE 0.42) in active rTMS group (Cohen's *d* = 0.93; *p* < .001) and 2.07 (SE 0.61) in sham rTMS group (Cohen's *d* = 0.65; *p* = .002). The improvements of mood and global cognitive function persisted for 8 weeks in both groups (active rTMS group: CSDD: Cohen's *d* = 0.74; *p* < .001; CES‐D: Cohen's *d* = 0.78; *p* < .001; HK MoCA: Cohen's *d* = 0.5; *p* < .001), but the magnitude of improvements declined over time, particularly in sham rTMS group (CSDD: Cohen's *d* = 0.49; *p* = .028; CES‐D: Cohen's *d* = 0.29; *p* = .068; HK MoCA: Cohen's *d* = 0.51; *p* = .012).

**TABLE 2:** Score changes of the treatment outcomes between the two randomized rTMS groups

	Active rTMS (*n* = 25)	Sham rTMS (*n* = 27)	*t*	*p*
Post‐intervention (T1)				
CSDD	3.24 ± 3.53	2.15 ± 3.22	1.17	0.25
CES‐D	3.61 ± 9.17	1.01 ± 5.66	1.24	0.22
HK MoCA	2.20 ± 1.94	1.89 ± 2.89	0.46	0.65
6th week follow‐up (T2)				
CSDD	2.84 ± 2.93	2.22 ± 2.94	0.76	0.45
CES‐D	2.72 ± 7.12	−0.07 ± 5.94	1.54	0.13
HK MoCA	2.20 ± 2.47	2.11 ± 2.83	0.12	0.90
12th week follow‐up (T3)				
CSDD	3.24 ± 2.83	2.56 ± 4.84	0.62	0.54
CES‐D	2.96 ± 6.82	1.18 ± 6.42	0.97	0.34
HK MoCA	3.04 ± 2.42	1.26 ± 3.37	2.17	0.03

*Note*: Data are raw scores and presented as mean ± SD.

Abbreviations: CES‐D, The Centre for Epidemiologic Studies Depression Scale; CSDD, Cornell Scale for depression in dementia; HK MoCA, The Montreal Cognitive Assessment Hong Kong version; rTMS, repetitive transcranial magnetic stimulation.

**FIGURE 3:**
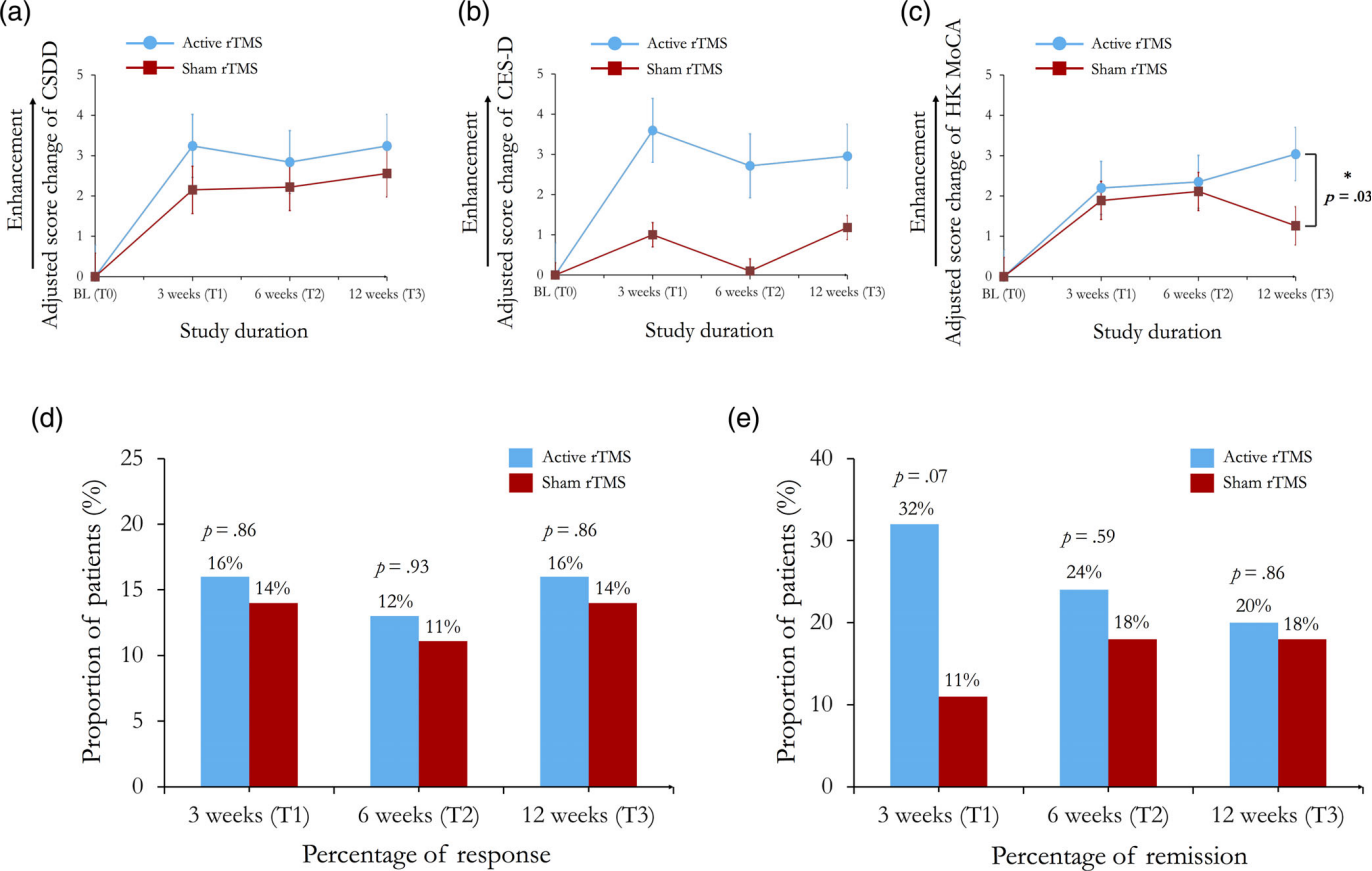
Primary outcome measures of the CSDD, the CES‐D and HK MoCA. The score changes of outcome measures from baseline to follow‐up time points were adjusted with baseline performance. Error bar represents the SEM. CES‐D, Centre for Epidemiologic Studies Depression Scale; CSDD, Cornell Scale for Depression in Dementia; HK MoCA, the Montreal Cognitive Assessment Hong Kong version

The changes of the total scores of CSDD and CES‐D did not differ between the two randomized groups across the follow‐up observations (Figure 3a,b). However, the NCD patients who received active rTMS had significant enhancements on mood‐relate signs (i.e., CSDD subitem 1) at T1 (*p* = .03) and T2 (*p* = .05) than the ones received sham rTMS (Table 3). The patterns of global cognition changes were similar in both groups at T1 and T2; while significant enhanced global cognition was only found in active rTMS group at T3 (*t* = 2.17, *p* = .03) (Figure 3c). The differences of clinical manifestations in the score changes of neuropsychiatric symptoms, quality of life and the incidence of adverse events were not observed between the two groups over the follow‐up periods.

**TABLE 3:** Score changes of the subitems of CSDD and CES‐D in the two randomized rTMS groups

	Active rTMS (*n* = 25)	Sham rTMS (*n* = 27)	*t*	*p*
Post‐intervention (T1)				
**CSDD1**	**1.25 ± 1.29**	**0.38 ± 1.39**	**2.28**	**.03**
CSDD2	0.13 ± 1.42	0.77 ± 1.27	−1.69	.10
CSDD3	0.42 ± 0.93	0.35 ± 1.06	0.25	.80
CSDD4	0.79 ± 2.00	0.35 ± 1.57	0.88	.38
CSDD5	0.63 ± 1.06	0.15 ± 1.19	1.48	.15
CES‐D1	1.29 ± 2.73	0.27 ± 1.91	1.55	.13
CES‐D2	0.17 ± 4.30	0.23 ± 3.43	−0.06	.95
CES‐D3	1.29 ± 4.23	0.50 ± 3.10	0.76	.45
CES‐D4	0.67 ± 2.43	0.04 ± 1.66	1.08	.29
6th week follow‐up (T2)				
**CSDD1**	**1.08 ± 1.59**	**0.27 ± 1.25**	**2.02**	**.05**
CSDD2	0.21 ± 1.35	0.77 ± 1.31	−1.49	.14
CSDD3	0.38 ± 1.06	0.35 ± 1.02	0.10	.92
CSDD4	0.75 ± 2.13	0.62 ± 1.88	0.24	.81
CSDD5	0.50 ± 0.93	0.15 ± 1.32	1.06	.29
CES‐D1	0.46 ± 2.73	−0.23 ± 2.86	0.87	.39
CES‐D2	1.00 ± 3.44	0.19 ± 2.99	0.89	.38
CES‐D3	0.92 ± 3.08	0.27 ± 2.41	0.83	.41
CES‐D4	0.33 ± 2.12	−0.31 ± 1.69	1.19	.24
12th week follow‐up (T3)				
CSDD1	1.25 ± 1.85	0.54 ± 1.39	1.53	.13
CSDD2	0.42 ± 1.10	0.81 ± 1.67	−0.97	.34
CSDD3	0.42 ± 1.06	0.04 ± 1.11	1.23	.23
CSDD4	0.67 ± 2.18	0.42 ± 1.98	0.41	.68
CSDD5	0.50 ± 0.98	0.27 ± 1.66	0.59	.56
CES‐D1	0.79 ± 2.45	0.69 ± 2.56	0.14	.89
CES‐D2	0.58 ± 3.30	−0.04 ± 3.07	0.69	.49
CES‐D3	1.17 ± 3.43	0.85 ± 2.92	0.35	.72
CES‐D4	0.33 ± 2.39	0.04 ± 1.73	0.50	.62

*Note*: Data are raw scores and presented as mean ± SD. Bold values are considered significantly different.

Abbreviations: CES‐D, The Centre for Epidemiologic Studies Depression Scale; CSDD, Cornell Scale for depression in dementia; rTMS, repetitive transcranial magnetic stimulation.

### 3.3 Response and remission rates

At the endpoint, the participants in active rTMS group had a 25.5% reduction in CSDD score compared with a 21.8% reduction in sham rTMS group (*p* = .75). Generally, the proportions of responders in active rTMS (4/25) were higher than those of sham rTMS (4/27) (*p* = .86), but the proportions of responders did not differ between the two randomized groups similarly over time (Figure 3d).

Furthermore, the proportion of participants achieving clinical remission was evaluated. Remission was defined by a total score of CSDD less than 7 at the time points following the completion of all rTMS treatment for each participant. For analytical purpose, participants from both groups were designated as remitters or non‐remitters. At T1, the proportion of remitters in active rTMS group (32%) were higher than that of sham rTMS group (11%) (*p* = .07), but the proportions of remitters did not differ between the two randomized groups at T2 and T3 (Figure 3e).

### 3.4 Measures of neuroplasticity

**FIGURE 4:**
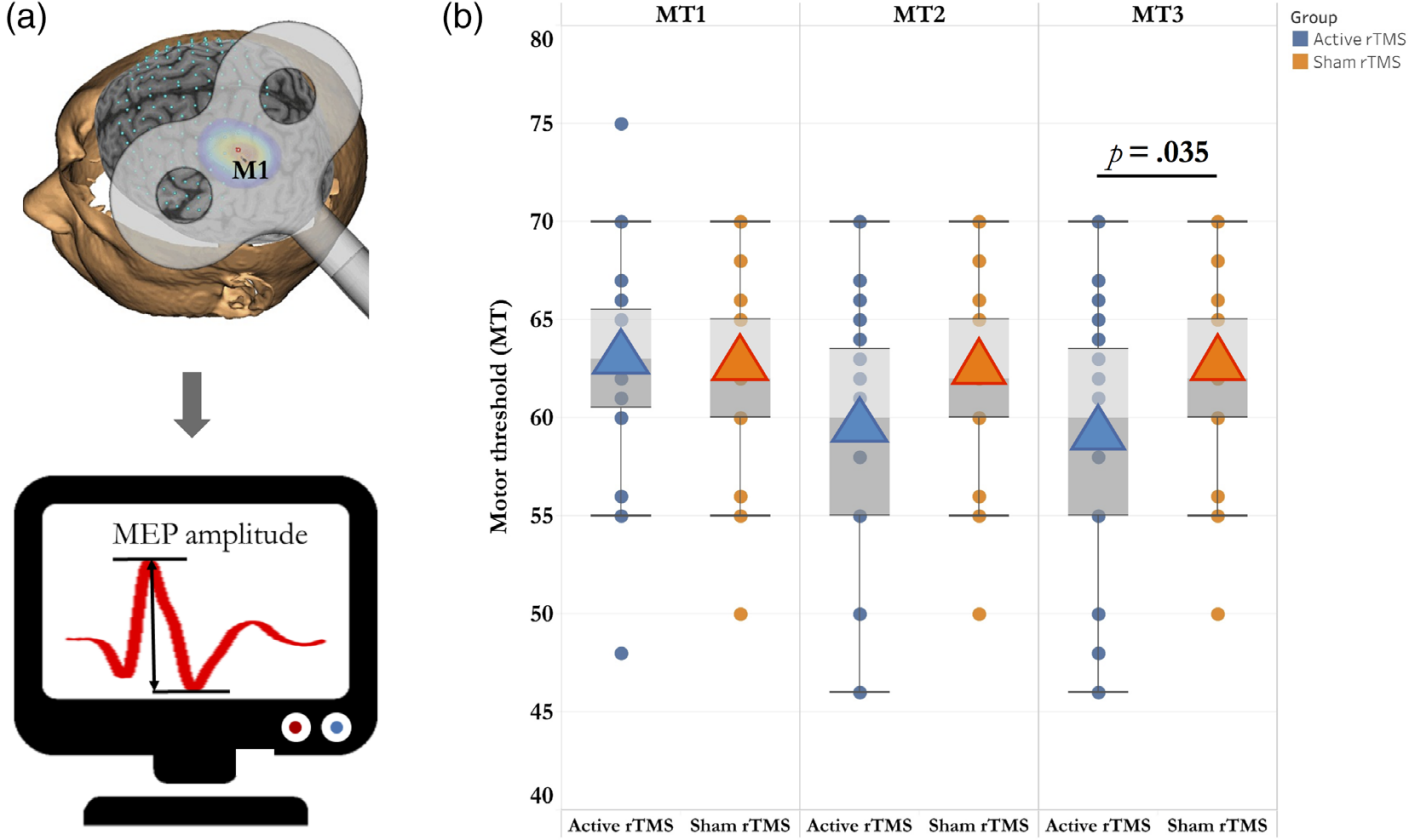
Comparisons of neuroplasticity measured by MT in active rTMS and sham rTMS groups. (a) The peak‐to‐peak amplitude of MEP induced by single pulse TMS over left primary motor cortex (M1) is used for determining the MT. (b) Box plot represents the differences of MT changes across time points. MEP, motor‐evoked potential; MT, motor threshold; rTMS, repetitive transcranial magnetic stimulation

After 5–10 sessions of rTMS treatment, a prominent decrease in motor threshold (MT) was found in active rTMS group (Figure 4b). Within each group, significant differences in the repeated measures of MT were observed in active rTMS group (MT1 vs. MT2: *t* = 3.014, *p* = .006; MT1 vs. MT3: *t* = 2.683, *p* = .013; MT2 vs. MT3: *t* = 1.001, *p* = .328), but not found in sham rTMS group (MT1 vs. MT2: *t* = 1.995, *p* = .067; MT1 vs. MT3: *t* = 1.443, *p* = .161; MT2 vs. MT3: *t* = −1.001, *p* = .327) (Appendix Table S1). Using age, sex and years of education as covariates, a significant association between MT changes and decreased CSDD total score was detected at T1 in active rTMS group (*r* = 0.548, *p* = .008).

At baseline, there were no associations between BDNF level, global cognition and the severity of depression. Compared with sham rTMS group, the BDNF level in active rTMS group demonstrated a marginally significant increase (*p* = .05) at T1. There was no group difference in the BDNF levels across all the time points (T0–T3) (Appendix Table S2). Using age and years of education as covariates, a weak but statistically non‐significant association between BDNF level and decreased CSDD total score was observed in active rTMS group (*r* = −0.677, *p* = .065) at T1, but not detected in sham rTMS group (*r* = −0.537, *p* = .136).

### 3.5 Differences of brain features

Two cases were excluded from this analysis due to severe cortical atrophy. With a corrected threshold of *p* < .05, no differences were observed in global grey matter volume (*t* = 0.959, *p* = .342), white matter volume (*t* = 1.136, *p* = .261), mean cortical thickness (*t* = −0.437, *p* = .664) and WMH (*t* = 0.041, *p* = .968) between active and sham rTMS groups (Appendix Table S3). The radiomic features of left DLPFC were calculated as grey matter volume (*t* = 0.703, *p* = .485), white matter volume (*t* = −0.415, *p* = .68), cortical thickness (*t* = 0.63, *p* = .531) and GI (*t* = 0.467, *p* = .642), which were comparable between the two randomized groups (Appendix Table S3).

### 3.6 Prediction performance of clinical and radiomic features

#### 3.6.1 Clinical prediction performance

As shown in Table 4, logistic regression analysis revealed that advanced chronological age (*p* = .016), higher baseline global cognition (*p* = .041) and group allocation (i.e., active rTMS) (*p* = .025) were associated with clinical remission at T1 (Nagelkerke *R* ^2^ = 0.42). Higher educational level could predict the clinical remission at T2 (OR = 0.82, *p* = .03) and T3 (OR = 0.756, *p* = .016) in active rTMS group. Group allocation was not a significant factor for clinical remission at T2 and T3.

**TABLE 4:** Estimated effect sizes for the independent variables included in the predictive models of rTMS treatments

	3rd week				6th week				12th week			
	OR	95% CI		*p*	OR	95% CI		*p*	OR	95% CI		*p*
		Lower	Upper			Lower	Upper			Lower	Upper	
Clinical variables												
Age (years)	0.891	0.795	0.998	**.047**	0.869	0.768	0.984	**.027**	1.036	0.912	1.176	.591
Sex	2.039	0.379	10.969	.407	0.865	0.127	5.893	.882	0.899	0.139	5.802	.911
Years of education	0.932	0.789	1.101	.407	0.820	0.685	0.981	**.03**	0.756	0.602	0.949	**.016**
Baseline HK MoCA score	0.773	0.6	0.995	**.041**	0.987	0.841	1.157	.868	0.851	0.685	1.058	.147
Radiomic variables												
Mean CT	6.072	0.599	61.532	.127	2.120	0.241	18.631	.498	9.517	0.867	104.436	.065
TIV	1	1	1	.846	1	1	1	.243	1	1	1	.923
GM volume of left DLPFC	1	1	1	.316	1	1	1	.833	1	1	1	.562
WM volume of left DLPFC	1	1	1.001	.384	1	1	1	.724	1	1	1	.792
CT of left DLPFC	0.031	0.001	0.819	**.03**	0.072	0.003	1.705	.103	1.108	0.103	11.946	.933
GI of left DLPFC	0.117	0.009	1.551	.104	3.937	0.062	250.873	.518	1.023	0.055	18.862	.988

*Note*: Data are raw scores and presented as mean ± SD.

Abbreviations: CI, confidence interval; CT, cortical thickness; DLPFC, dorsolateral prefrontal cortex; GI, gyrification index; GM, grey matter; HK MoCA, the Montreal Cognitive Assessment Hong Kong version; OR, odds ratio; rTMS, repetitive transcranial magnetic stimulation; TIV, total intracranial volume; WM, white matter.

#### 3.6.2 Radiomics prediction performance

**FIGURE 5:**
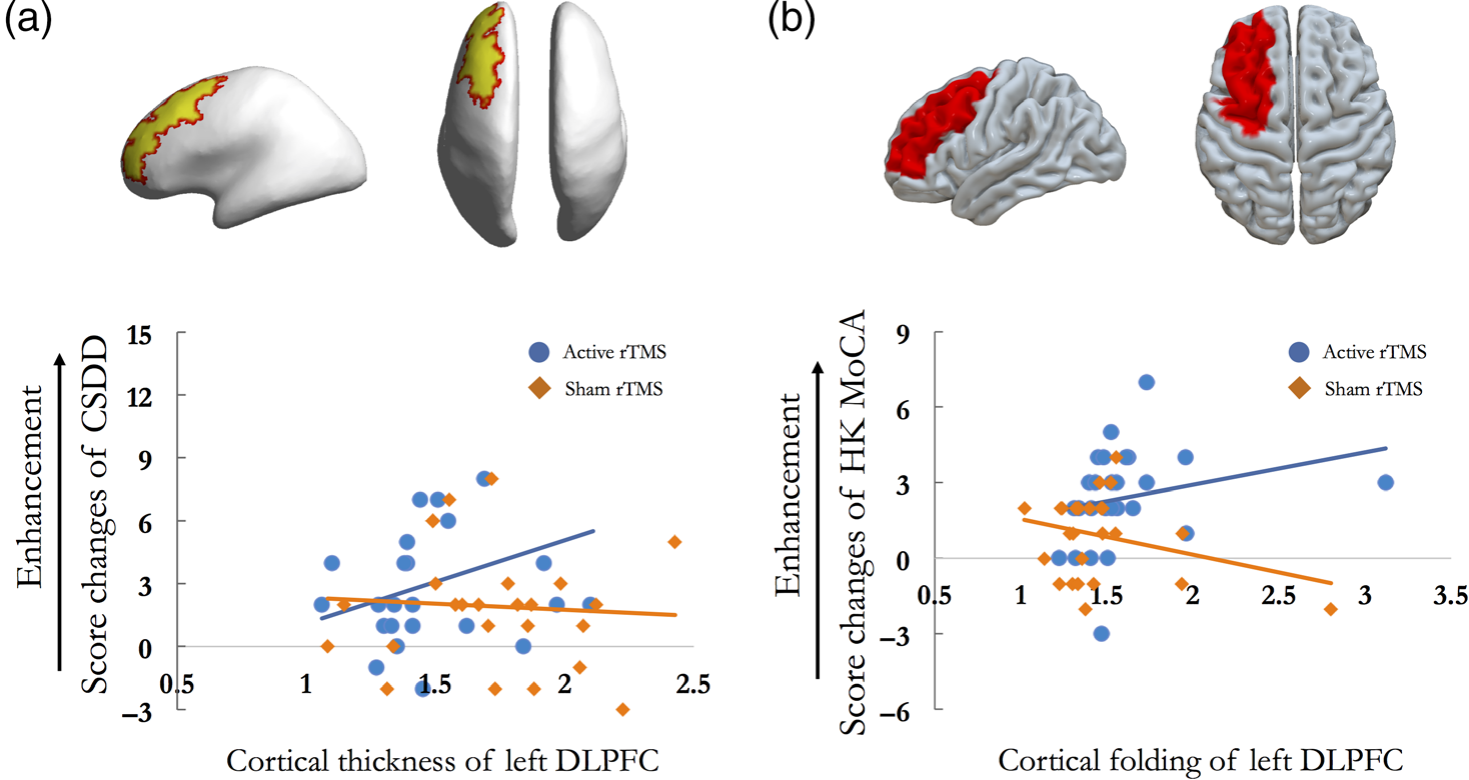
Radiomic features response likelihoods of patients treated with rTMS. (a) Increased cortical thickness of left DLPFC could predict the mood enhancement measured by the CSDD in active rTMS group at T1. (b) Increased cortical folding of left DLPFC could predict the cognitive improvement measured by HK MoCA in active rTMS group at T1. CSDD, Cornell scale for depression in dementia; DLPFC, dorsolateral prefrontal cortex; HK MoCA, the Montreal Cognitive Assessment Hong Kong version; rTMS, repetitive transcranial magnetic stimulation

We found no significant effects of the mean cortical thickness and TIV on the chance to achieve clinical remission when controlling for the demographic variables included in the model. In contrast, treatment target‐specific morphometric features, cortical thickness and folding in particular, demonstrated to be the significant predictors to predict the clinical remission and cognitive improvements. Increased cortical thickness of left DLPFC could predict the clinical remission in the NCD patients received active rTMS at T1 (OR = 0.31, *p* = .03, 95%CI: 0.001–0.819) (Figure 5a). Increased cortical folding (measured by GI) of left DLPFC could predict the cognitive improvements in the patients received active rTMS at T1 (OR = 0.318, *p* = .038, 95%CI: −4.989 to −0.148) (Figure 5b).

## 4 DISCUSSION

In this clinical trial, we investigated the safety, efficacy and sustainability of MRI‐guided 10 Hz rTMS over left DLPFC for alleviating depressive symptoms and cognitive impairment in patients with major NCDs. A 3‐week course of 10 Hz rTMS treatment, either active rTMS or sham rTMS, had a positive effect on mood and global cognitive function, but the NCD patients who received active rTMS had a higher remission rate than the ones received sham rTMS. The baseline clinical features in terms of age, years of education and global cognition were associated with better treatment outcomes. Moreover, the longitudinal regression model that included the treatment target‐specific (i.e., left DLPFC) radiomic features showed the substantially higher odds of better outcomes compared to the model that included the whole‐brain MRI features. The findings indicate that the quantitative measures of treatment target‐specific cortical features are valuable to pursue and apply in TMS studies.

The antidepressant effects of 10 Hz rTMS have been demonstrated in the treatment of mood disorders in different age groups (George et al., 2000; Pascual‐Leone et al., 1996) and the patients with neurological disorders (Schutter, 2009). When treating patients with old age, prominent reductions in depressive symptoms were observed after rTMS treatment (Gu & Chang, 2017; Jorge et al., 2008), but the response rate and remission rate in elderly patients were not consistent across the published studies. For instance, Figiel et al. (1998) found that 10 Hz rTMS could produce a modest antidepressant effect in elderly patients with a response rate of 23%, which was lower than that of young patients (56%). Later, a similar result was obtained, but a higher response rate was observed in elderly patients (45%) (Fabre et al., 2004). Interestingly, the antidepressant effects of rTMS have been challenged by the results from sham‐controlled trials. For instance, Mosimann et al. (2004) reported that the efficacy of active rTMS was not superior to sham rTMS for treating depression in elderly patients.

Indeed, although both active and sham rTMS showed significant antidepressant effects with a medium to large effect size (i.e., 0.66–0.92), the magnitude of effect size in active rTMS group was higher that of sham rTMS group. The observed effect size in this clinical trial is aligned with, and even larger than the ones reported in previous studies (Brys et al., 2016; Jorge et al., 2008). It is interesting to notice that the comparable antidepressant effects of rTMS between the two randomized groups were not reflected in clinical effectiveness. While not significant, the remission rates in patients received active rTMS were nearly three times higher than those received sham rTMS (i.e., 32% vs. 11%). After 15 sessions of rTMS, although the magnitude of alleviating depressive symptoms was gradually diminished over time, the significant enhancements on mood‐relate signs (i.e., CSDD subitem 1) in active rTMS group suggest that group allocation is still a key factor related to the positive treatment outcomes in major NCD with depression.

Upon the antidepressant effects of 10 Hz left DLPFC rTMS, another interesting finding of this clinical trial is a synergistic effect on global cognitive function across the follow‐up periods. In fact, it is not surprising to observe the depressive symptoms reduction along with enhanced cognitive functions (Fabre et al., 2004), because of the strong relationship between the two conditions. The shared neuronal basis of cognitive impairment and depression could be explained from the perspectives of structural and functional imaging. Left DLPFC, as part of neocortex, plays a critical role in high‐level cognitive functions and mood regulation in cognitive disorders and depression. Ageing and neurodegeneration is also associated with the structural and functional changes of left DLPFC. For example, the cortical reductions and metabolic hypoactivity of left DLPFC have been observed in normal ageing adults (Salat et al., 2004) and Alzheimer's disease patients (Liang et al., 2011). Findings from functional MRI also revealed the abnormal connections between left DLPFC and other brain regions at resting state or during task performance are related to poor cognitive performance (Toepper, 2017; Zheng et al., 2019). The left DLPFC connectivity could serve as a neural marker of cognitive reserve, reflecting the flexibility in brain networks involved in complex cognitive functions in preclinical AD patients (Franzmeier et al., 2017). Meanwhile, neuroimaging studies have confirmed that left DLPFC was underactive (Baxter et al., 1989; Fitzgerald et al., 2008) and the connections between DLPFC and other cortical or subcortical regions (e.g., parietal cortex, anterior cingulate) were disturbed in people with depression (Li et al., 2018). The TMS over left DLPFC can increase the activities of DLPFC and related functional connectivity in people with depression (Struckmann et al., 2022).

Even though the phenomenon of synergistic effects has been reported by a few TMS studies (Jodoin et al., 2019; Koch et al., 2020), the dissociable trajectories of cognitive and mood changes in active and sham rTMS groups indicate that rTMS may have differential effects on cognitive functions and mood regulation characterized with neuroplasticity. For instance, on the one hand, although the serum BDNF level in active rTMS group only showed a marginally significant increase compared with sham rTMS group, no noticeable associations were detected between baseline BDNF level, severity of depression and cognitive functions. On the other hand, the significant decreased motor threshold after rTMS treatment and its associations with the amelioration of depressive symptoms in active rTMS may be explained by the potential neuroplasticity induced by active rTMS.

The prediction performance of baseline demographic and clinical features on treatment outcomes was limited, with only educational level remaining as a significant predictor in the regression models. Although age strongly related to positive post‐intervention outcomes, as well as in previous studies (Berlim et al., 2014; Conelea et al., 2017), it did not significantly predict long‐term clinical outcomes. Based on the cognitive reserve hypothesis, a higher level of education is thought to reflect a higher level of cognitive reserve and possibly protects against the accumulating brain pathology and related cognitive impairment during ageing (Stern, 2012). The presence of a positive effect of education on the short‐term outcomes for NCD patients can possibly indicate that this protective effect is largely continued and most relevant to the treatment‐induced neural plasticity. Interestingly, baseline global cognition did not differ between the two randomized groups, but still can predict the clinical remission in NCD patients received active rTMS, which suggests that a quick assessment of a patient's global cognition, as a component of cognitive reserve, might be valuable in predicting the likelihood of positive outcomes to the treatment. At baseline, instead of whole‐brain MRI features, the treatment target‐specific radiomic features turned out to be the significant predictive markers in the remitters. Especially pre‐treatment thicker cortex could predict a higher probability of clinical remission; and increased cortical folding could predict a higher cognitive enhancement after active rTMS treatment. While previous imaging studies already reported the predictive values of whole‐brain MRI measures (Schwarz, 2021), the treatment target‐specific radiomic features are more likely involved in the complex interface between the cortex and the stimulation‐induced electric fields (Aberra et al., 2020; Lu, 2022), which is valuable to in‐depth investigate in future studies.

## 5 CONCLUSION

To sum up, a 3‐week 10 Hz rTMS over left DLPFC showed significant treatment effects on mood and global cognition in individuals with co‐occurring depression and NCDs, but the ones who received active rTMS had a higher remission rate than the ones received sham rTMS. The findings give some supports for considering 10 Hz rTMS as an adjuvant treatment for its rapid antidepressant and cognitive‐enhancing effects bridging the therapeutic gap when medications take a longer time to take effect on mood and cognition. Pre‐treatment radiomics, including cortical thickness and gyrification of the treatment target, were significant MRI‐informed predictors of clinical remission and cognitive enhancement in individuals received active rTMS treatment.

## 6 LIMITATIONS AND FUTURE DIRECTIONS

Findings of this study should be carefully interpreted in the context of its limitations. Most notably, without the detailed information of the subtypes of NCDs, we are unable to evaluate the effectiveness of rTMS in disease‐specific populations. For instance, individuals with NCD‐AD and NCD‐vascular may have the similar clinical symptoms of depression and cognitive deficits, which are due to different etiologies and neuropathologies. To better control the baseline clinical features within the participants, we compared the scores of cardiovascular risks, white matter volume and WMH (i.e., vascular effects on brain) and found no differences between active and sham rTMS group.

Another important limitation is the treatment duration and TMS dosing applied in this study. Compared to the standard‐of‐care FDA‐approved TMS protocol for treating major depressive disorder, our protocol only applied half of the total number of TMS pulses. Although our main purpose was to examine the rapid antidepressant and cognitive effects of rTMS in NCD patients, the shortened treatment duration and reduced TMS pulses may weaken the modulating effect and related neural plasticity induced by active rTMS. An additional limitation was the use of medication. It is difficult to control and quantify the influence of medications that the participants routinely take to treat their chronic diseases during the study period. Although no differences in the medication regimes and dosing were found in the participants received active or sham rTMS, there might be potential effects of medication on the response to rTMS that could not be evaluated separately. Finally, without the follow‐up assessments of structural MRI, we are unable to detect the dynamic brain changes and the predictive values of radiomic features for the treatment response to rTMS.

For future studies, it would be informative to explore and establish better TMS‐specific parameters (i.e., target location, treatment duration and magnetic intensity) to improve the TMS treatment for disease‐specific populations. Meanwhile, it will be imperative to examine the neurophenotypes for the prediction of treatment response at individual level. For example, radiomics of treatment targets, including cortical features (i.e., cortical thickness and folding) and transcranial features (i.e., scalp‐to‐cortex distance and thickness of CSF), should be involved for constructing the personalized TMS model for real‐world study. Furthermore, the long‐term effects of standard TMS or novel modalities of TMS (i.e., theta‐burst stimulation) on neural plasticity, resilience and maintenance of mood and cognition would be in‐depth investigated in future practice.

### ACKNOWLEDEGMENTS

The authors are grateful to the research staff at the Chan Wei Wei Therapeutic Physical Mental Exercise Centre and the Department of Imaging and Interventional Radiology. We gratefully acknowledge the study investigators and the dedication of all the participants and their families.

## AUTHOR CONTRIBUTIONS

Linda Chiu Wa Lam and Sandra Sau Man Chan conceived the study. Lin Shi and Defeng Wang helped to conduct the MRI scanning. Ma Sukling conducted the BDNF assessment. Vincent Chung Tong Mok and Arthur Dun‐Ping Mak helped to recruit the participants. Lin Cui Chan helped to conduct the assessments. Hanna Lu developed the hypotheses, analyzed the imaging data, conducted the rTMS treatment and wrote this article.

## FUNDING INFORMATION

This work was supported by the Hong Kong Research Grant Council (RGC)—General Research Fund (GRF) (Grant No. 14111115).

## CONFLICT OF INTEREST

The authors have declared that no competing interests.

## Supporting information


**Table S1** Measurements of motor threshold in the two randomized rTMS groups

**Table S2** Measurements of serum BDNF in the two randomized rTMS groups

**Table S3** Measurements of global brain morphometry and radiomics of left DLFPC in the two randomized rTMS groups

## Data Availability

The demographic, clinical and raw imaging data used and analyzed in this study are available from the corresponding authors on reasonable request.
