# Supplementary material for: Clinical and radiomic features for predicting the treatment response of repetitive transcranial magnetic stimulation in major neurocognitive disorder: Results from a randomized controlled trial
Source: Hum Brain Mapp. 2022 Aug 1;43(18):5579–92. doi: 10.1002/hbm.26032 (PMC9704797; doi:10.1002/hbm.26032)
Supplement: Supplementary file 1 — Table S1 Measurements of motor threshold in the two randomized rTMS groups [file HBM-43-5579-s003.docx]

Appendix table 3. Measurements of global brain morphometry and radiomics of left DLFPC in the two randomized rTMS groups

|  | Active rTMS | Sham rTMS | *t* value | *p* value |
| --- | --- | --- | --- | --- |
| Global brain morphometry |  |  |  |  |
| Global GM volume (mm^3^) | 417923.99±53707.73 | 405907.88±45781.92 | 0.959 | 0.342 |
| Global WM volume (mm^3^) | 258304.31±58885.53 | 242272.91±39814.73 | 1.136 | 0.261 |
| Mean cortical thickness (mm) | 3.63±0.41 | 3.73±0.45 | -0.437 | 0.664 |
| PVH | 1.45±0.69 | 1.41±0.62 | 0.171 | 0.865 |
| WMH | 1.27±0.79 | 1.29±0.77 | -0.071 | 0.944 |
| WMH total score | 2.73±1.42 | 2.71±1.31 | 0.041 | 0.968 |
| Radiomics of left DLPFC |  |  |  |  |
| GM volume (mm^3^) | 12013.31±2602.91 | 11521.84±2483.29 | 0.703 | 0.485 |
| WM volume (mm^3^) | 7278.48±1949.12 | 7493.10±1817.14 | -0.415 | 0.680 |
| Cortical thickness (mm) | 3.79±0.47 | 3.64±0.49 | 0.630 | 0.531 |
| Gyrification index | 1.58±0.36 | 1.54±0.31 | 0.467 | 0.642 |

Note. Data are raw scores and presented as mean ± SD.

Abbreviations: DLPFC = Dorsolateral prefrontal cortex; rTMS = Repetitive transcranial magnetic stimulation; GM = Grey matter; WM = White matter; PVH = Periventricular hyperintensity; DWMH = Deep white matter hyperintensity; WMH = White matter hyperintensity.
