# Supplementary material for: Clinical and radiomic features for predicting the treatment response of repetitive transcranial magnetic stimulation in major neurocognitive disorder: Results from a randomized controlled trial
Source: Hum Brain Mapp. 2022 Aug 1;43(18):5579–92. doi: 10.1002/hbm.26032 (PMC9704797; doi:10.1002/hbm.26032)
Supplement: Supplementary file 2 — Table S2 Measurements of serum BDNF in the two randomized rTMS groups [file HBM-43-5579-s001.docx]

Appendix table 1. Measurements of motor threshold in the two randomized rTMS groups

| Time points | Motor threshold (MT) | | | |
| --- | --- | --- | --- | --- |
|  | Active rTMS | Sham rTMS | *t* value | *p* value |
| MT1 | 63.08 ± 5.41 | 61.96 ± 5.06 | 0.78 | 0.453 |
| MT2 | 59.92 ± 6.11 | 61.77 ± 4.93 | -1.19 | 0.242 |
| MT3 | 59.42 ± 6.57 | 61.88 ± 5.12 | -1.49 | 0.143 |

Note. Data are raw scores and presented as mean ± SD.

Abbreviations: rTMS = Repetitive transcranial magnetic stimulation; MT = Motor threshold.
