# Supplementary material for: Clinical and radiomic features for predicting the treatment response of repetitive transcranial magnetic stimulation in major neurocognitive disorder: Results from a randomized controlled trial
Source: Hum Brain Mapp. 2022 Aug 1;43(18):5579–92. doi: 10.1002/hbm.26032 (PMC9704797; doi:10.1002/hbm.26032)
Supplement: Supplementary file 3 — Table S3 Measurements of global brain morphometry and radiomics of left DLFPC in the two randomized rTMS groups [file HBM-43-5579-s002.docx]

Appendix table 2. Measurements of serum BDNF in the two randomized rTMS groups

| Time points | Brain-derived neurotrophic factor (BDNF) | | | |
| --- | --- | --- | --- | --- |
|  | Active rTMS | Sham rTMS | *t* value | *p* value |
| Baseline | 7608.94 ± 5924.78 | 8315.96 ± 5720.79 | -0.38 | 0.704 |
| 3^rd^ week FU | 10252.06 ± 7559.28 | 10419.02 ± 6308.11 | -0.07 | 0.942 |
| 6^th^ week FU | 9323.46 ± 6206.85 | 8808.79 ± 6379.83 | 0.25 | 0.808 |
| 12^th^ week FU | 11719.82 ± 9734.67 | 12235.51 ± 5860.73 | -0.21 | 0.839 |

Note. Data are raw scores and presented as mean ± SD.

Abbreviations: BDNF = Brain-derived neurotrophic factor; rTMS = Repetitive transcranial magnetic stimulation; FU = Follow-up.
